# Mapping mechanical properties of organic thin films by force-modulation microscopy in aqueous media

**DOI:** 10.3762/bjnano.3.53

**Published:** 2012-06-26

**Authors:** Jianming Zhang, Zehra Parlak, Carleen M Bowers, Terrence Oas, Stefan Zauscher

**Affiliations:** 1 Department of Mechanical Engineering and Materials Science, Duke University, Durham, North Carolina 27708, USA; 2 Center for Biologically Inspired Materials and Materials Systems, Duke University, Durham, North Carolina 27708, USA; 3 Department of Biochemistry, Box 3711, Duke University Medical Center, Durham, North Carolina 27710, USA; 4 Department of Chemistry, Duke University, Durham, North Carolina 27708, USA

**Keywords:** acoustic atomic force microscopy, biomolecules, elastic modulus mapping, nanomechanical characterization, self-assembled monolayers

## Abstract

The mechanical properties of organic and biomolecular thin films on surfaces play an important role in a broad range of applications. Although force-modulation microscopy (FMM) is used to map the apparent elastic properties of such films with high lateral resolution in air, it has rarely been applied in aqueous media. In this letter we describe the use of FMM to map the apparent elastic properties of self-assembled monolayers and end-tethered protein thin films in aqueous media. Furthermore, we describe a simple analysis of the contact mechanics that enables the selection of FMM imaging parameters and thus yields a reliable interpretation of the FMM image contrast.

## Introduction

Mapping the mechanical properties, such as elastic modulus, friction, and adhesion of surfaces and thin films in aqueous (or liquid) environments with nanoscale lateral resolution is important for a broad range of applications in materials science [1–10] and in the life sciences [11–20]. The atomic force microscope (AFM) [21], due to its force sensitivity and ability to image surface topography with high lateral resolution, is ideally suited to map these properties. Intermittent AFM imaging modes, such as tapping mode [22–24], and pulsed-force mode [12,25–28], have been developed for soft, often biological, samples in liquid environments. Although these imaging modes reduce the lateral forces, they often do not allow direct interpretation of the data in terms of the surface mechanical properties, due to cantilever damping in solution and the complex forces that the probe experiences when jumping in and out of contact with the surface.

Alternatively, dynamic variations of contact mode AFM, such as acoustic AFM, add a small actuation to the tip–surface contact at acoustic frequencies and are thus useful for mapping differences in the surface mechanical properties of the sample [29]. In some versions of acoustic AFM, such as ultrasonic AFM (UAFM) [30], acoustic force atomic microscopy (AFAM) [31], and contact resonance AFM (CR-AFM) [32–35], contact resonance frequencies are deliberately chosen to enhance the imaging sensitivity. However, acoustic AFM imaging in solution is challenging since the liquid phase complicates the cantilever dynamics through fluid damping. To our knowledge, only a few studies report the use of acoustic AFM on molecularly thin films or soft materials in liquid [7,36].

Here we show that force-modulation microscopy (FMM) is a powerful acoustic AFM method for mapping surface mechanical properties in fluids. In a typical FMM setup, the tip–sample contact is actuated at an off-resonance frequency, and the amplitude and phase response of the cantilever vibration are then detected at the drive frequency, by using a lock-in amplifier, and mapped concurrently with topography [37]. The narrow detection bandwidth used in FMM entails less noise, while off-resonance actuation reduces fluid-related cantilever dynamics. Consequently, FMM can map even slight differences in the sample surface stiffness (i.e., the contact stiffness). While these advantages were shown in some FMM studies performed on monolayers [38–39], the understanding of amplitude and phase contrasts and the frequency limitations of FMM in liquid, remain incomplete, which often leads to conflicting data interpretation [38–39]. Presently, these unresolved issues diminish the usefulness of FMM as a mechanical mapping tool in materials science, especially for molecular thin films and biological samples.

In this article, we describe the use of FMM for mapping subtle differences in the elastic properties of organic thin films in aqueous environments. To this end we developed a parameter selection method for FMM that helps (i) in the selection of appropriate actuation frequencies and contact forces, and (ii) in the unambiguous interpretation of the contrast in the amplitude images [38–40]. We demonstrate the capability of FMM to image mechanical properties in aqueous media on surface-tethered proteins and self-assembled EG_3_-thiol (triethylene glycol mono-11-mercaptoundecyl ether) monolayers. Our studies show that subtle differences in the packing order of the self-assembled EG_3_-thiols manifest as differences in the surface elastic properties that can be mapped by FMM in solution. The results presented in this paper also provide a stepping stone for the development of a quantitative viscoelastic modeling approach in liquids, in analogy to those developed for contact resonance AFM in air [32–33].

## Results and Discussion

### FMM working principles

#### Linear regime in FMM

In FMM, the cantilever tip contacts the substrate surface with a constant static force while a small force modulation is superimposed [37]. As a first approximation, this contact can be modeled by Hertzian contact theory. Though based on the assumption of a nonadhesive and elastic contact between a rigid spherical tip and the substrate surface, the model readily and adequately explains contact mechanics when the static contact force is much greater than the adhesion force [41–43]. Furthermore, the Hertzian contact model has been successfully extended to characterize the stiffness of thin, layered materials [3,44]. If necessary, tip–sample adhesion can easily be included in the contact analysis by selecting an appropriate contact mechanics model, such as the Johnson–Kendall–Roberts (JKR) or the Derjaguin–Muller–Toporov (DMT) model [41,45].

Although contact deformation and force have a nonlinear relationship in the Hertzian contact model, this model can be linearized for a small force modulation at high contact forces, and the stiffness of the contact can be determined [46–47]. Linearization is valid as long as the cantilever is in constant contact with the sample and the amplitude of the force modulation is much smaller than the contact force.

For a lossless contact and for modulation frequencies significantly below the contact resonance frequency, the cantilever and the contact can be modeled as two springs in series (see Supporting Information File 1). In summary, the deflection of the cantilever, *u*_c_, measured by FMM is,

[1]
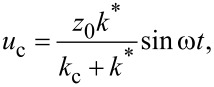


where *z*_0_ is the actuation amplitude of the contact, ω is the angular frequency of the actuation, *k*_c_ is the spring constant of the AFM cantilever, and k^*^ is the contact stiffness,

[2]
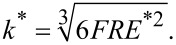


The contact stiffness is a function of the reduced Young’s modulus, E^*^, the tip radius, *R*, and the applied force, *F*.

Equation 1 explains how the amplitude of the AFM cantilever deflection is related to *z*_0_, *k*_c_ and k^*^. Since *z*_0_ and *k*_c_ do not change while the surface is being scanned, *u*_c_ depends only on k^*^. The cantilever vibration amplitude is thus smaller on soft regions (low k^*^), and it is higher on stiff regions (high k^*^). Although this simple analysis provides a convenient explanation of the contrast mechanism in FMM amplitude images, Equation 1 cannot be used to quantify FMM experiments [48], because the modulation frequency is typically not sufficiently low that the cantilever dynamics can be ignored.

#### Nonlinear regime in FMM

The current understanding of FMM is largely based on the amplitude and phase response of the cantilever at large static loading forces and very small modulation amplitudes. Imaging of compliant samples, however, requires overall low contact forces in combination with a high modulation amplitude for sensitive mapping. This combination precludes linearization of the contact models. For this case of soft contact, the sinusoidal force modulation at a single frequency yields a nonlinear (distorted sinusoidal) cantilever deflection response, which reflects the contact nonlinearity and gives rise to higher harmonics, as shown in Equation 3 (see also Supporting Information File 1).

The cantilever deflection with a second-order harmonic can be rewritten as,

[3]
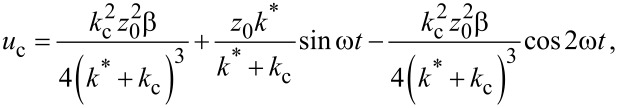


where 
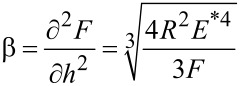
 is the second-harmonic factor.

The frequency-independent, zeroth-order term in Equation 3 reflects a DC deflection. The feedback loop, however, cannot differentiate this zeroth-order component from the surface-topography-induced deflection response of the cantilever, thus precluding clear signal deconvolution [29]. Both the first and second harmonics, however, do not interfere with the feedback loop and can be detected by lock-in techniques. At low forces, the second-harmonic factor (β) increases dramatically, and thus promotes the contribution from the second-harmonic amplitude.

**Figure 1:**
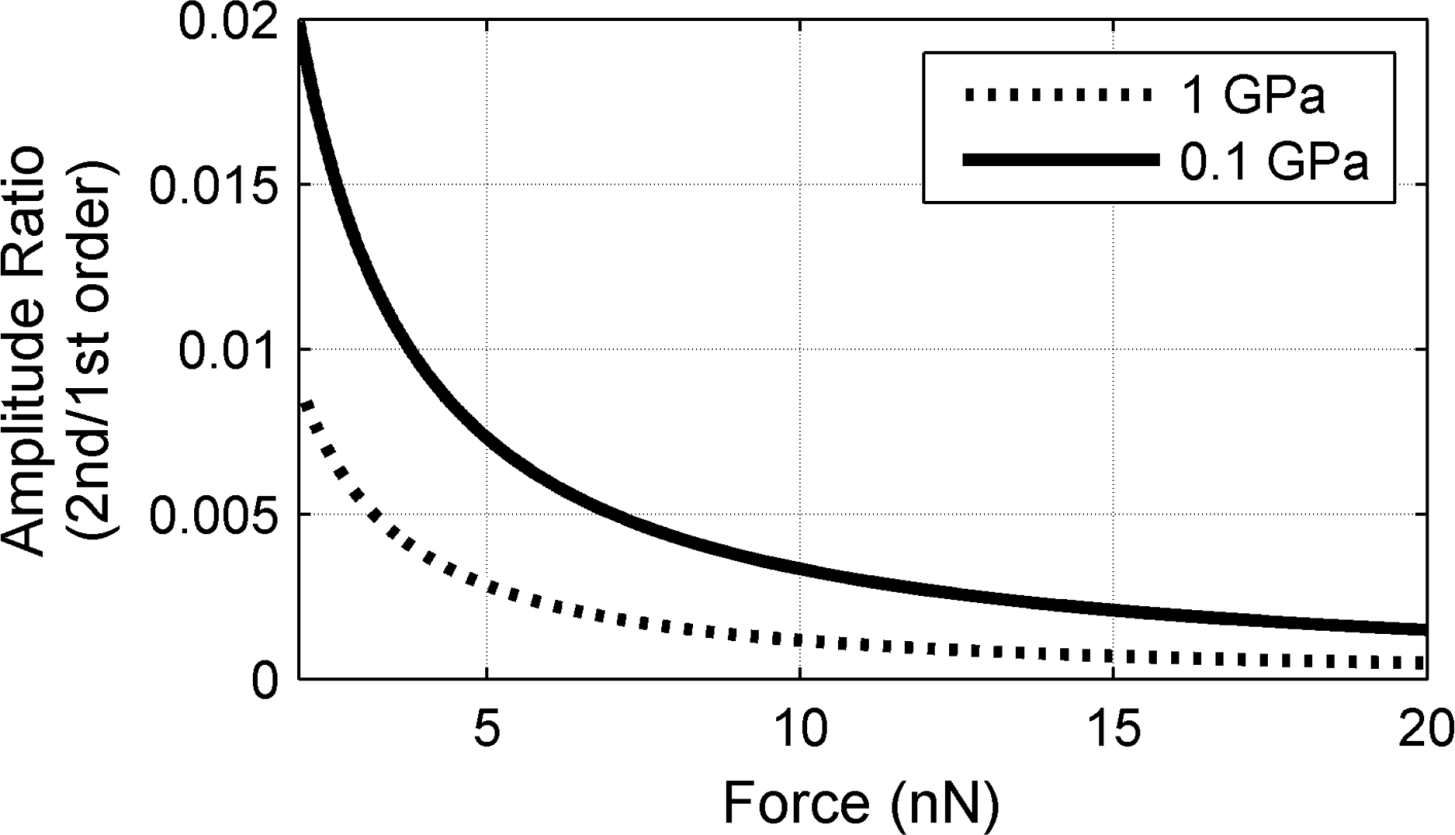
Amplitude ratio of the second to the first harmonic, plotted for different applied forces. The surface modulus is set to 0.1 GPa (solid) and to 1.0 GPa (dotted).

The ratio of the second- to first-harmonic amplitudes is plotted in Figure 1 as a function of contact force for two reduced moduli. This ratio was calculated by using realistic experimental parameters, i.e., *k*_c_ = 1 N/m, *R* = 30 nm, and *z*_0_ = 2 nm, while 0.1 GPa and 1 GPa were assigned to E^*^. FMM measurements are less nonlinear at (i) high contact forces and (ii) for stiff materials, as shown by the lower amplitude ratio in these cases in Figure 1. This implies that changes in the surface elasticity can lead to nonlinear effects in FMM, making a quantitative interpretation of the amplitude and phase signals complicated, especially at low applied forces.

#### Parameter selection and contrast interpretation for hard-contact FMM in aqueous environments

The interpretation of FMM amplitude and phase images obtained on soft substrates is further complicated by viscous damping effects [49], particularly when imaging in an aqueous environment. To better interpret image contrast in that case, one needs to understand the dependence of amplitude and phase on surface stiffness, and one needs a method to select the proper contact force and actuation frequency. Here we use contact force as a variable to change the contact stiffness (Equation 2) and monitor the response of the amplitude and phase behavior of the cantilever.

**Figure 2:**
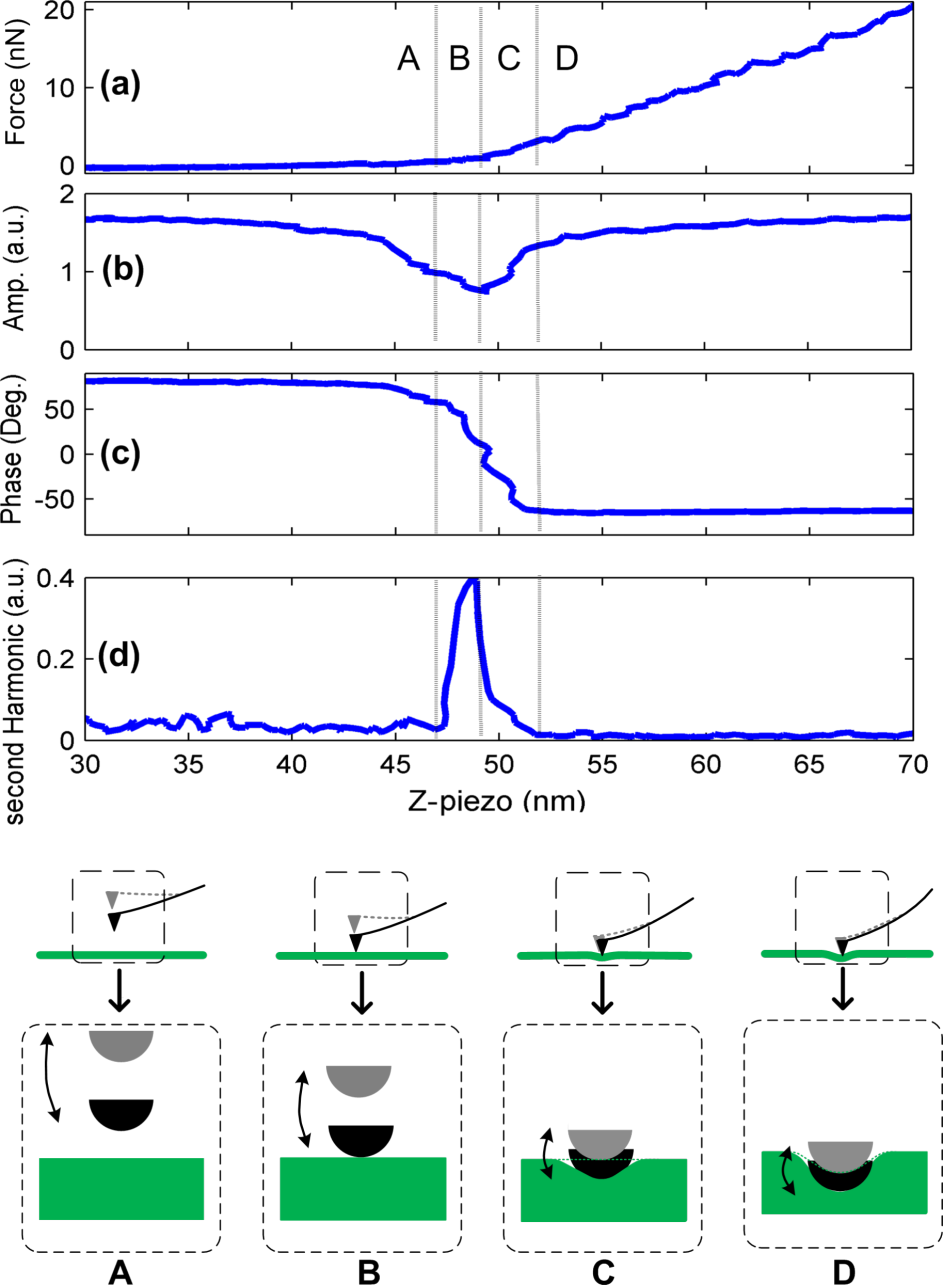
(a) Force, (b) first-harmonic amplitude, (c) first-harmonic phase, and (d) second-harmonic amplitude, plotted as a function of Z-piezo displacement. As the probe approaches the gold sample surface, the cantilever encounters four regimes: (A) free oscillation, (B) partial contact, (C) soft contact, and (D) hard contact. To highlight the differences in cantilever bending and the level of indentation in the four regimes, the schematic is not drawn to scale.

In our parameter-selection process we acquire force–distance curves while the cantilever is modulated at the desired frequency. We monitor (i) the amplitude and (ii) the phase of the first harmonic, and (iii) the amplitude of the second harmonic of the cantilever oscillations, along with (iv) the cantilever deflection, as the cantilever interacts with the surface (Figure 2). The deflection of the cantilever determines the interaction force from which the contact stiffness can be calculated (Equation 2). The amplitude of the first harmonic is used to analyze the elasticity of the substrate surface in FMM and it is thus essential to relate the first harmonic with the contact stiffness experimentally. Meanwhile, the amplitude of the second harmonic, a measure of the nonlinearity in the contact, should be minimized for reliable FMM measurements. A set of representative curves for cantilever deflection, first-harmonic amplitude and phase, and second-harmonic amplitude, at 20 kHz actuation frequency in water on a gold surface, are shown in Figure 2. For these experiments, we used a cantilever with a spring constant of 0.9 N/m and a resonance frequency of 47.8 kHz in solution.

The different regions of the deflection (Figure 2a) and amplitude curves (Figure 2b and Figure 2d) indicate both the position of the probe and the type of the contact. In regime A the cantilever freely oscillates with a zero mean deflection; however, the amplitude decreases slightly with decreasing tip–sample distance. Because the amplitude of the second harmonic (Figure 2d) is still small [24,50], this behavior can likely be attributed to hydrodynamic lubrication forces that increase with increasing proximity of the tip to the surface [49]. In regime B, the amplitude of the first harmonic decreases, while that of the second harmonic increases, reflecting the increasing nonlinearity of the initial tip–surface interaction and the change in cantilever dynamics, when the cantilever approaches the surface. In regime C, the amplitude of the first harmonic of the cantilever vibration increases, while that of the second harmonic decreases. This behavior is consistent with the analytical expressions for the soft contact (Figure 1) [29,37]. When hard contact is reached in regime D, the contact force and the amplitude of the first harmonic are high, whereas the amplitude of the second harmonic is close to zero again. We note that both regimes, A and D, have high amplitudes for the first harmonic. This is quite different from the behavior in tapping-mode AFM, in which the amplitude in regime A is typically much larger than that in regime D [51]. In tapping-mode AFM, the cantilever is intentionally actuated at its resonance frequency to achieve a large cantilever amplitude. In FMM, however, the actuation frequency is typically well below the free resonance frequency, and the actuation amplitude is selected to yield a small cantilever amplitude in contact. Furthermore, as shown in Figure 2, AFM tip–surface interactions should be kept in regime D to obtain a linear contact response. This demand needs to be balanced by the need for low applied forces that are required to image compliant samples nondestructively. Consequently, the boundary between regimes C and D determines the minimum applicable contact force for which a sufficiently linear vibration response is obtained. To demarcate the onset of regime D, we have chosen the ratio of the first- to the second-harmonic amplitudes to be less than 0.1% (0.001). The first harmonic vibration amplitude increases with increasing contact force in regimes C and D, indicating that higher contact stiffness values (see Equation 2) cause higher amplitudes. On the other hand, increasing the contact stiffness decreases the phase response (Figure 2c). As a consequence, soft regions on the sample appear bright in the phase images. Importantly, however, the higher phase observed on softer areas reflects the convolution of the cantilever dynamics and time-dependent contact stiffness, and is thus not a result of the substrate viscoelasticity alone. The force, amplitude, and phase measurements shown in Figure 2 were carried out on thin gold surfaces whose apparent stiffness can be represented by a simple spring. Even in this simple case, a quantitative description of the cantilever dynamics in aqueous solution is complicated and not yet available. However, the measurements shown in Figure 2 can help to understand how the cantilever responds to changes in surface stiffness (for a given set of FMM imaging parameters).

To account quantitatively for the viscoelastic mechanical properties of soft polymeric and biomolecular thin films, requires the inclusion of a viscoelastic model, such as the Voigt model, to explain the tip–sample interaction. Such an approach has recently been shown for contact-resonance imaging in air [32]. However, as before, the cantilever dynamics, which depends not only on fluid loading but also on the details of the applied force (see above), needs to be captured adequately first, before a meaningful deconvolution of the contact stiffness is possible.

Another issue concerns the selection of the actuation frequency in FMM. Force–distance curves recorded at different actuation frequencies show that when actuation is above the free resonance frequency of the cantilever, higher forces are required to establish hard contact (regime D in Figure 2). This is due to the fact that the contact of the tip with the surface changes the cantilever dynamics and increase the resonance frequency. Consequently, the cantilever modulation increases and contact nonlinearity occurs. In this case, a simple correlation between contact stiffness and first-harmonic amplitude can lead to conflicting results [52]. To avoid this situation, one should select an actuation frequency far below the free resonance frequency of the cantilever.

#### FMM on patterned protein monolayers

Characterizing the dynamic mechanical properties of biomolecular monolayers provides insight into the dynamics of biomolecules on surfaces and aids in the design of functional biomolecular micro- and nanostructures. Here, acoustic AFM methods are promising tools since they enable sensitive mapping of the contact-mechanical properties of samples by introducing high-frequency modulation while imaging the topography [53]. Although these methods have been used in air, imaging of many polymers and biomolecules should take place in an aqueous environment or under physiological conditions. Here we show that FMM is able to provide high-contrast amplitude and phase maps of micropatterned biomolecular thin films in an aqueous environment.

**Figure 3:**
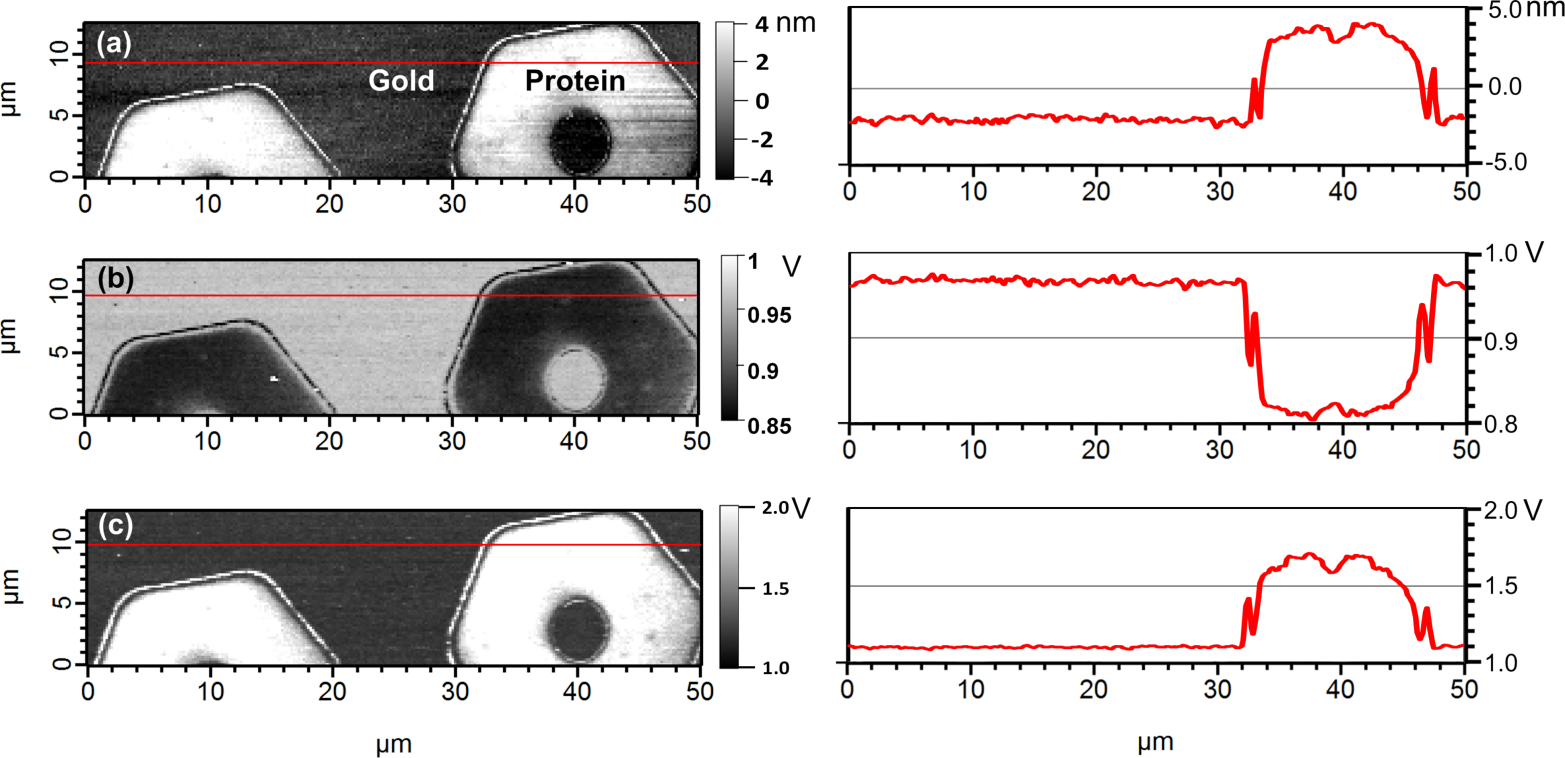
FMM images of SpA-N B-domain protein patterns on a gold surface, with corresponding cross-section analysis along the red line in the AFM images. (a) The height image shows a protein height of about 5 nm. (b) The amplitude and (c) phase images of the same area clearly show the elastic difference between protein and the gold substrate.

The biological material of interest in our FMM experiments is the IgG-binding domain of staphylococcal protein A. Protein A is a surface protein found on the cell wall of *staphylococcus aureus* bacteria and contains five domains for IgG-binding (SpA-N). One of the domains is named the B-domain and its structure and folding behavior have been well studied [54]. Specifically, we use FMM to image and map differences in the elastic properties of micropatterned, end-tethered proteins (constructs of five repeating SpA B-domains) on gold. The topography, amplitude, and phase images were obtained in PBS buffer at 35 kHz actuation frequency with 9 Å vibration amplitude and 8 nN contact force (Figure 3), which leaves the cantilever and surface in hard contact (region D in Figure 2).

The dark regions in the amplitude image indicate that the contact stiffness (and thus largely the protein sample) is considerably softer than the gold substrate. The FMM height image (Figure 3a) shows that the protein layer is approximately 5 nm thick. The corresponding amplitude image at an excitation frequency of 35 kHz (Figure 3b) shows that protein regions have about 17% lower amplitude than the gold substrate (as shown in the cross-section). This suggests, as anticipated, that the protein patterns are significantly softer than the gold substrate. Force–distance curves on the gold and protein regions showed that the adhesion force between the AFM probe and the protein features is negligibly small. The adhesion force on gold is around 0.3 nN, which is only about 3% of the static force applied, while the adhesion force on the protein surface is within the noise level of the measurement. This justifies the use of a Hertzian contact mechanics model, as done here.

Our approach currently does not capture the viscoelasticity of the protein or the response of the cantilever to a viscoelastic contact in aqueous solution. Future work to quantify these properties requires additional analytical models that capture the interaction of the cantilever beam with the liquid environment.

#### FMM on patterned EG_3_-thiol monolayers

The properties and applications of alkanethiol self-assembled monolayers (SAMs) on gold surfaces have been the subject of interface science research for many years. The self-assembly of alkane thiol molecules on gold surfaces is a two-step process. The initial physisorption step on gold substrates is typically slow and concentration-dependent [55]. Once in contact, the molecules adsorb on the gold substrate in a loosely packed configuration, with the thiol end binding to gold and the carbon chain aligning approximately parallel to the surface [56–57]. The persistence of this stage depends on the thiol concentration, and thus on the initial packing density and order of the thiol molecules on the surface. At low concentrations, this lying-down phase can persist for hours. At high concentrations, however, thiols can reorient into an upright conformation and pack tightly on the surface within seconds. The adsorption process has been studied with several surface-sensitive techniques, including surface plasmon resonance (SPR) [58], quartz-crystal microbalance (QCM) [59–61] and ellipsometry [62]. These methods, however, do not resolve differences in the grafting density and packing of the molecules with high spatial resolution (micrometer or less). Here we show that FMM in solution is able to distinguish subtle difference in the packing of self-assembled thiol monolayers on surfaces, by mapping the amplitude of the first harmonic of the cantilever vibration amplitude.

**Figure 4:**
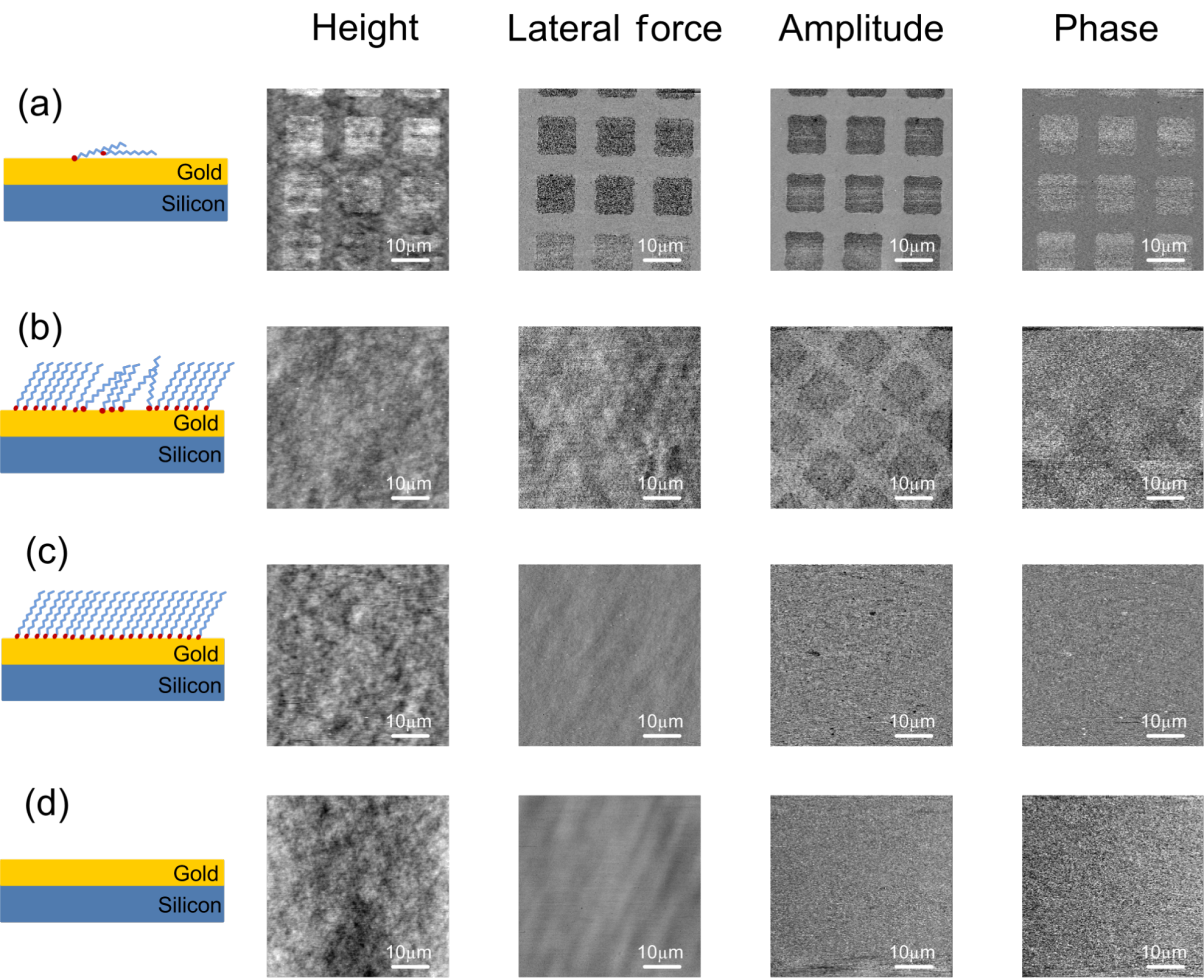
Schematic and FMM images of a series of EG_3_ patterns on gold. Height, lateral force, amplitude, and phase images were captured simultaneously. (a) Low-EG_3_-grafting-density areas patterned on a gold substrate. The height difference between EG_3_ and gold is about 1 nm. The lateral-force image shows the chemical force difference between the areas. The contrast in the amplitude image demonstrates that the EG_3_ areas are significantly softer than the gold background. (b) Low-EG_3_-grafting-density areas (squares) and high-grafting-density background. Height and lateral-force images cannot resolve differences in the morphology and chemical force, while amplitude images differentiate between high- and low-grafting-density regions (i.e., the original patterns are visible in the amplitude image). (c) Patterned surfaces imaged after overnight exposure to a thiol solution. Height, chemical-force, and stiffness images are uniform. (d) Negative control: gold surface after photolithography and resist stripping shows no surface residues.

Figure 4 shows a series of FMM images of patterned EG_3_-thiols (triethylene glycol mono-11-mercaptoundecyl ether, HO(CH_2_CH_2_O)_3_C_11_H_22_SH) obtained at 20 kHz. The patterns were prepared by photolithography. Briefly, the sample was prepared by immersing the developed photoresist pattern in a 10 µM thiol solution for 60 s, followed by stripping with ethanol and washing with Milli-Q grade water (see Experimental section for details). The sample was backfilled with thiol molecules at high concentration for different lengths of time. An EG_3_-thiol SAM is about 2.4 nm thick when thiols are in a close-packed 
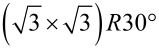
 configuration, while the thickness is only about 0.4 nm when the thiol chains lie flat on the surface [63].

The first row of FMM images in Figure 4a were obtained simultaneously on an EG_3_-patterned sample. From left to right, the images are height, lateral force, and amplitude and phase of the first harmonic of the cantilever vibration, respectively. The height of the EG_3_-thiol patterns is 1.7 ± 1.1 nm, which suggests that the thiol molecules are not close-packed, and have some disorder in their arrangement on the surface. The contrast in the lateral-force image shows a friction difference between the gold surface and the EG_3_ patterns that can be attributed to the surface-energy difference between the ethylene glycol end groups and the gold [64]. The low amplitude and high phase of the first-harmonic signal on the thiol patterns indicate that the regions covered by EG_3_ molecules are softer than the gold substrate.

The first harmonic amplitude curves obtained from force–distance measurements, reflect the apparent stiffness of the EG_3_ layer (see Supporting Information File 1 for details) [65]. The apparent Young’s modulus of the thiols on the surface is around 30 GPa, consistent with moduli of short alkanethiol chains obtained by using SEM and nano-indentation [66–67]. The approach to deconvolute these Young’s moduli further to reflect the layered systems of thiol SAMs on gold substrates has been shown in the literature [68], but is beyond the scope of this discussion.

The second row (Figure 4b) shows images obtained on a sample first patterned by exposure to 10 µM EG_3_-thiol for 1 min, followed by stripping off of the photoresist, and backfilling in 0.5 mM EG_3_-thiol for 1 h. Both height and friction images do not show any pattern-related contrast, which suggests that the molecules have a similar height and the same surface chemical properties. Importantly, however, the original patterns become clearly visible in the amplitude image, and somewhat less clearly in the phase image. The contrast in the amplitude image shows that the patterned areas are “softer” than the likely more-ordered regions that are backfilled at high thiol concentrations. This result suggests that FMM detects the subtle elastic differences between the patterned and backfilled regions.

We ascribe the contrast in the FMM amplitude and phase images to differences in the packing order of the thiols on the substrate surface. The thiol SAMs assembled in the second step by backfilling with thiol solutions at high concentrations, form a standing-up phase on bare gold almost immediately [56,69]. At the same time, in areas that were previously self-assembled with thiols, the reorientation of thiols is slower than that in the backfilled areas, which would entail an overall less-ordered conformation. Our results not only illustrate the effect of grafting density and molecular packing on the apparent layer stiffness, but also demonstrate the high sensitivity of FMM in solution for imaging self-assembled monolayers.

The third row (Figure 4c) shows images obtained on a sample first patterned by exposure in 10 µM EG_3_-thiol for 1 min, followed by stripping of residue resist and overnight exposure to 0.5 mM EG_3_-thiol. As shown previously, the packing of thiols on a surface equilibrates to a well-ordered layer with overnight thiol exposure [70–71]. Our data are in agreement with this notion, as we did not observe any surface morphological or mechanical differences in the AFM images. The elimination of the differences could be caused by the long-time equilibration, which leaves the surface with a uniformly ordered layer of thiol molecules. The last row (Figure 4d) shows FMM images obtained on a control sample (bare gold, after photoresist stripping), processed in parallel, but without thiol deposition. The height, lateral force, and amplitude and phase images do not show any difference in the morphology or the substrate mechanical properties, suggesting that the photoresist developing and stripping steps did not change the surface properties.

## Conclusion

We showed that force-modulation microscopy (FMM) can be used to image organic thin films in aqueous environments with high spatial resolution and sensitivity to conformational details that affect the contact mechanics. FMM generated high-contrast amplitude and phase images of proteins end-grafted to gold substrates, and reflects the expected (see Equation 1) differences in contact-stiffness on the sample. Furthermore, FMM experiments on self-assembled thiol monolayers were highly sensitive to differences in the surface elastic properties arising from subtle differences in the molecular packing of the thiols on the substrate surface.

Although previous FMM studies observed the contrast in amplitude and phase images [9,37–38,72], the interpretation of the results was inconsistent because the relation between the contrast mechanism and the cantilever dynamics was not sufficiently considered [38,48], particularly in aqueous environments. We thus developed a parameter-selection procedure that allows for reliable interpretation of image data, and accounts for the effect of contact force and actuation frequency on the cantilever dynamics in FMM. More specifically, this procedure determines the minimum contact force necessary, at a certain excitation frequency, to establish a linear response in the contact regime.

## Experimental

### FMM setup

**Figure 5:**
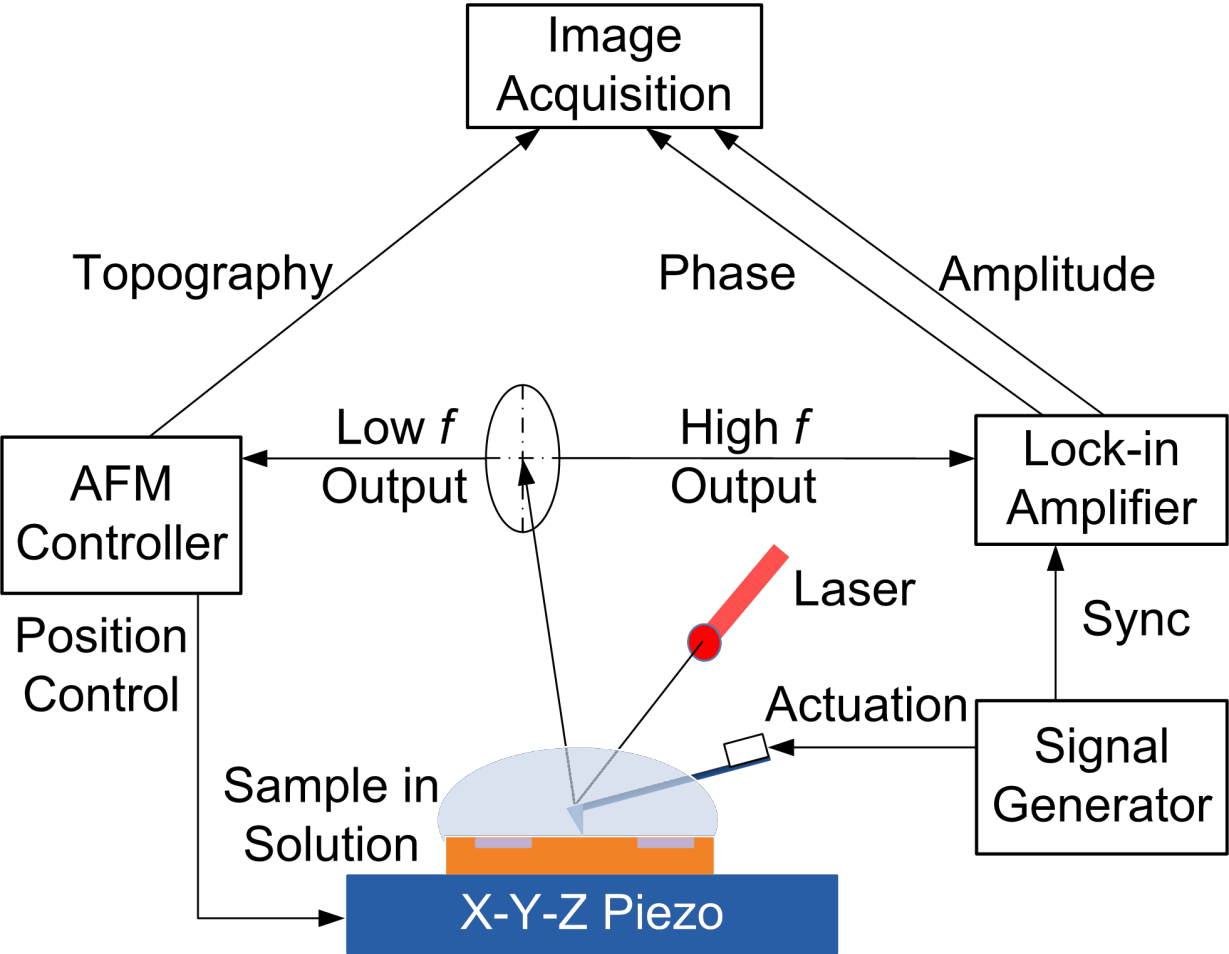
Schematic of the FMM setup. The AFM probe is kept at a constant static contact force when scanning the sample in solution. The signal generator actuates the cantilever probe with a single frequency signal, and the cantilever response is monitored by a lock-in amplifier.

A commercial AFM system (Asylum MFP-3D) was modified to implement FMM [37,72] in liquid as shown in Figure 5. Like in contact-mode imaging, the feedback controller of the AFM keeps the tip–sample force constant during the surface scan. In addition, however, a piezoelectric transducer in the cantilever holder was used to excite the cantilever with a small amplitude, off-resonance frequency. A lock-in amplifier (AMETEK model 7280) was used to monitor the amplitude and phase of the resulting cantilever vibration at the actuation frequency.

All samples were imaged at a rate of 1 line/s and at a resolution of 256 pixels per line. The actuation frequency of the cantilever was kept higher than 8 kHz to avoid interference with the AFM imaging feedback control. A cantilever in contact has contact resonance modes [46] and the cantilever vibration amplitude is amplified at the contact resonance frequency, which increases with increasing surface stiffness. Contact resonances modes in air have been used to quantify the stiffness of surfaces [31]. However, the quality factor of these modes decreases significantly in solution and makes it difficult to interpret cantilever vibrations around contact resonance modes. A proper probe for FMM imaging in liquid should have a high resonance frequency to simplify data analysis and at the same time it should be soft to prevent destructive forces on compliant samples. Therefore we used ScanAsyst-Fluid cantilevers (Bruker Probes) that have 0.7 N/m nominal spring constant and 50 kHz free resonance frequency in solution. The deflection sensitivity of each cantilever was determined from a force–displacement curve taken before an FMM experiment. The spring constant of each cantilever was calculated from the power spectral density of the thermal noise fluctuations.

Since FMM is a modified contact mode AFM method, frictional forces may affect the measurements. Friction leads to lateral twisting of the cantilever, which may be coupled with the actuation normal to the contact. To decrease the effect of friction on the amplitude and phase images of FMM, the slow scan direction is selected perpendicular to the cantilever axis. Meanwhile, the use of triangular cantilevers minimizes the torsional twisting of the cantilever.

### Sample Preparation

#### Gold deposition

Silicon wafers (Virginia semiconductor, Part 325S119656) were washed in acetone, ethanol and DI water, and completely dried before use. A 45 nm gold layer with a 5 nm chromium adhesion layer was deposited on the silicon surface by using an e-beam thermal evaporator (Kurt Lesker PVD 75), and subsequently cleaned by ozone plasma ashing (Emitech K-1050X).

#### Protein monolayer

Five tandem B-domains of staphylococcal protein A were expressed and purified from *E. coli*. The C-terminus of the terminal protein was modified with cysteine to enable protein binding to the gold surface. Protein patterns were prepared by dry stamping of the tandem B-domains on to the gold substrate surface, by using a polyurethane (pUA) stamp (15 µm hexagon). The pUA stamp was UV cross-linked on a silicon master with hexagonal pattern features and, before each use, cleaned by UV–ozone exposure. For dry stamping, 100 μL of a 500 μM protein solution was inked on the pUA surface and incubated for 10 min, followed by drying in a stream of nitrogen. The stamp was then brought into contact with a cleaned gold surface for 30 s. The patterned surface was subsequently sonicated and rinsed in deionized (DI) water followed by nitrogen drying.

#### Patterned EG_3_-thiol monolayers

**Figure 6:**
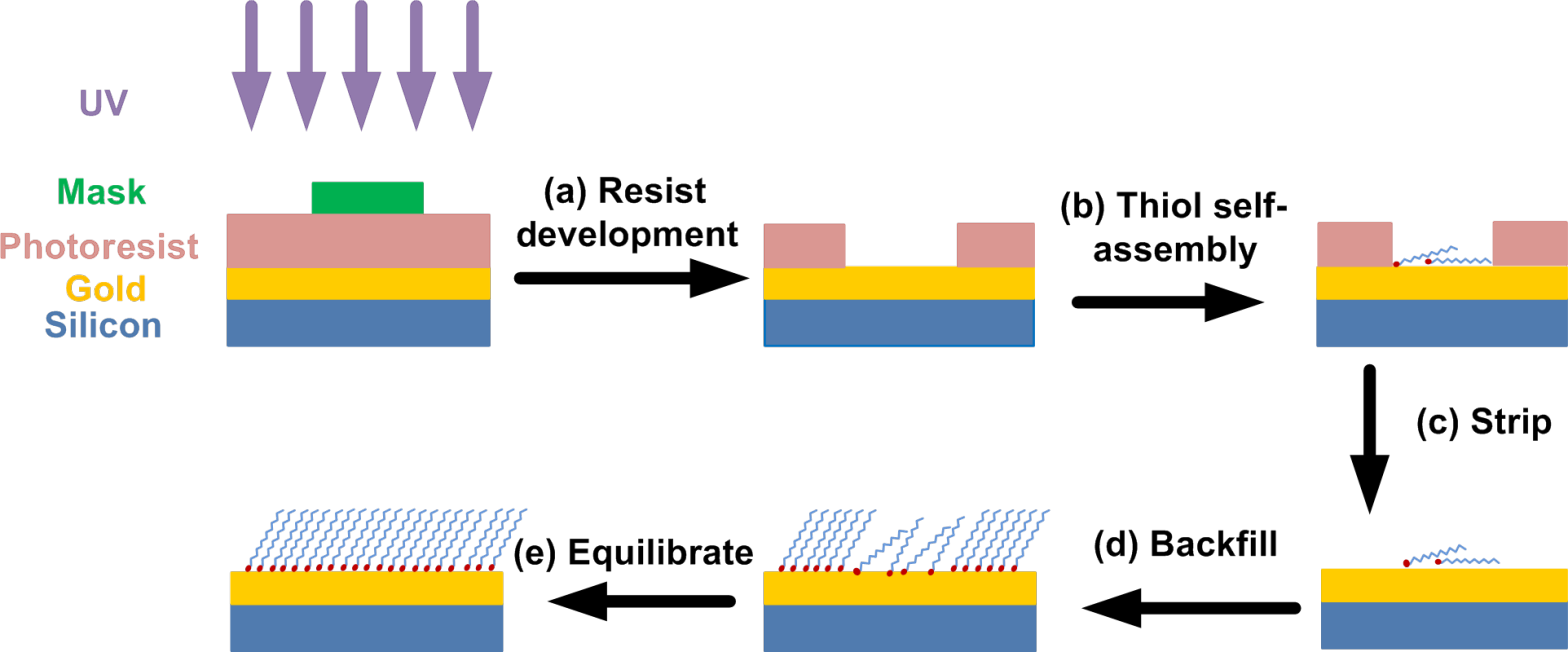
Schematic of the photolithography process for EG_3_-thiol pattern deposition. (a) A micropatterned gold surface, covered with a negative tone resist, is patterned by exposure to UV light through a photomask. (b) Self-assembly of EG_3_ thiols at low concentration generates low-grafting-density patterns. (c) Residual negative resist is stripped off by solvent washing. (d) Newly exposed gold surface is covered by high-grafting-density EG_3_ thiol by backfilling. (e) Overnight exposure to EG_3_ solution equilibrates the patterned-thiol SAM to a uniform surface.

A 3 µm thick layer of negative tone resist (NFR-016D2) was spin-coated onto a freshly deposited and cleaned gold surface at 3000 rpm (Figure 6). A photolithography mask was then used to create 8 × 8 µm^2^ square patterns during UV exposure. Next, the exposed photoresist was removed (Figure 6a), and the wafer was then cut into 1 × 1 cm^2^ squares, which were rinsed in 0.5% SDS solution and DI water, and dried under N_2_. The substrate chips were then exposed for 60 s to a solution of 10 µM EG_3_-thiol (triethylene glycol mono-11-mercaptoundecyl ether, HO(CH_2_CH_2_O)_3_C_11_H_22_SH) in 2% ethanol (Figure 6b), followed by rinsing with copious amounts of DI water and drying in a stream of nitrogen. This treatment produced EG_3_-thiol patterns with low grafting density. Next, the remaining negative photoresist was stripped by acetone sonication for 1 min and ethanol wash (Figure 6c). The whole surface was then exposed to 0.5 mM ethanolic EG_3_-thiol solution for different lengths of time to generate different thiol packing densities on the substrate surface (Figure 6d). Thiol adsorption on the bare gold surfaces occurs at high solution concentrations, the thiol grafting density is high, and the molecules are in an upright conformation. With prolonged exposure to high thiol solution concentrations, the grafting density and packing of the molecules equilibrates by backfilling and exchange reactions, and becomes eventually indistinguishable from the background. By varying the reaction time and thiol concentration in the solution phase, thiol patterns with two different packing orientations were generated on the gold substrate surface.

## Supporting Information

The cantilever response in linear and nonlinear contact regimes is derived in more detail.

File 1 Force modulation of the cantilever response.
