# Supplementary material for: Mapping mechanical properties of organic thin films by force-modulation microscopy in aqueous media
Source: Beilstein J Nanotechnol. 2012 Jun 26;3:464–74. doi: 10.3762/bjnano.3.53 (PMC3458590; doi:10.3762/bjnano.3.53)
Supplement: File 1 — Force modulation of the cantilever response. [file Beilstein_J_Nanotechnol-03-464-s001.pdf]

Supporting Information

for

Mapping mechanical properties of organic thin films by force-modulation microscopy in aqueous media

Jianming Zhang[1,2,§], Zehra Parlak[1,2,3,§], Carleen M. Bowers[4], Terrence Oas[3], and Stefan Zauscher[*1,2]

Address: [1]Department of Mechanical Engineering and Materials Science, Duke University, Durham, North Carolina 27708, USA, [2]Center for Biologically Inspired Materials and Materials Systems, Duke University, Durham, North Carolina 27708, USA, [3]Department of Biochemistry, Box 3711, Duke University Medical Center, Durham, North Carolina 27710, USA and [4]Department of Chemistry, Duke University, Durham, North Carolina 27708, USA

Email: Prof. Stefan Zauscher - zauscher@duke.edu

[*] Corresponding Author

[§] These authors contributed equally to the paper

Force modulation of the cantilever response

The contact between the AFM tip and the surface can be modeled by Hertzian contact theory. In Hertzian contact models, the applied contact force, F, has the following relation with the indentation (h):

$$F = \frac{4}{3} E^* \sqrt{R} h^{\frac{3}{2}}, \tag{1}$$

where R is the tip radius and E^* is the reduced Young's modulus. E^* is a function of the Young's moduli of the surface and the tip (E_s and E_t), and the Poisson ratio of the surface and the tip (ν_s and ν_t),

$$E^* = \left(\frac{1-\nu_t^2}{E_t} + \frac{1-\nu_s^2}{E_s} \right)^{-1}. \tag{2}$$

In FMM, the contact of the tip and the surface is modulated, while a higher contact force is applied. One can apply a Taylor series expansion to Equation 1 to determine indentation modulation, δ, for the added force modulation (F_{ac}):

$$F_{ac} = \frac{\partial F}{\partial h}\delta + \frac{1}{2}\frac{\partial^2 F}{\partial h^2}\delta^2 + \frac{1}{6}\frac{\partial^3 F}{\partial h^3}\delta^3 \ldots \quad . \tag{3}$$

To stabilize the system, the contact force equilibrates with the force on the cantilever which can be expressed by using the cantilever deflection and the stiffness (k_c):

$$F_{ac} = k_c \left(z_0 \sin \omega t - \delta \right), \tag{4}$$

where z_0 is the amplitude of the actuation and ω is the radial frequency of the actuation. Since the sample surface is indented with the applied modulation, the cantilever deflects as much as u_c, the difference between the modulation and the indentation:

$$u_c = z_0 \sin \omega t - \delta. \tag{5}$$

When the force modulation is much smaller than the contact force, the first term of Equation 3 is higher than other terms and the contact can be modeled by a linear spring. The stiffness of this spring is called the contact stiffness, k^*:

$$\frac{\partial F}{\partial h} = k^* = \sqrt[3]{6FRE^{*2}}. \tag{6}$$

However, the second- and higher-order terms become more prominent when low contact forces are applied. The quadratic term in Equation 3 is

$$\frac{\partial^2 F}{\partial h^2} = \beta = \sqrt[3]{\frac{4R^2E^4}{3F}}. \tag{7}$$

1. Linear contact regime

For small modulation amplitudes and high contact forces, the first term of Equation 3 is enough to capture the force modulation, and the force equilibrium can be written as

$$F_{\mathrm{ac}} = k^*\delta = k_{\mathrm{c}}\left(z_0 \sin \omega t - \delta\right). \tag{8}$$

The cantilever deflection, u_{c}, can then be reduced to the following expression:

$$u_{\mathrm{c}} = \frac{k^*}{k^* + k_{\mathrm{c}}} z_0 \sin \omega t. \tag{9}$$

2. Nonlinear contact regime

Low contact forces are desired for imaging compliant samples but this causes the second and higher orders of Equation 3 to rise. To understand the force modulation with small nonlinearity, let's assume that the second term in Equation 3 is sufficient. In this case, the force equilibrium is

$$F_{\mathrm{ac}} = k^*\delta + \frac{1}{2}\beta\delta^2 = k_{\mathrm{c}}\left(z_0 \sin \omega t - \delta\right). \tag{10}$$

When the quadratic equation in Equation 10 is solved, δ can be deduced:

$$\delta = \frac{k^* + k_c}{\beta}\left(-1 \pm \sqrt{1 + \frac{2k_c \beta z_0 \sin \omega t}{\left(k^* + k_c\right)^2}}\right). \tag{11}$$

Since a negative δ is not physically meaningful, the positive root is selected and the square root is expanded in a second-order Taylor series:

$$\begin{aligned}\delta &= \frac{k^* + k_c}{\beta}\left(-1 + 1 + \frac{k_c \beta z_0 \sin \omega t}{\left(k^* + k_c\right)^2} - \frac{\left(k_c \beta z_0 \sin \omega t\right)^2}{2\left(k^* + k_c\right)^4}\right) \\ &= \frac{k_c z_0 \sin \omega t}{k^* + k_c} - \frac{k_c^2 \beta z_0^2 \left(\sin \omega t\right)^2}{2\left(k^* + k_c\right)^3}.\end{aligned} \tag{12}$$

The cantilever deflection shown in Equation 5 can then be obtained by using this nonlinear indentation:

$$u_c = \frac{k^*}{k^* + k_c} z_0 \sin \omega t + \frac{k_c^2 \beta z_0^2 \left(\sin \omega t\right)^2}{2\left(k^* + k_c\right)^3}. \tag{13}$$

Equation 13 can be rewritten by using trigonometric identities:

$$u_c = \frac{k_c^2 \beta z_0^2}{4\left(k^* + k_c\right)^3} + \frac{k^*}{k^* + k_c} z_0 \sin \omega t - \frac{k_c^2 \beta z_0^2 \cos\left(2\omega t\right)}{4\left(k^* + k_c\right)^3}. \tag{14}$$
